# Effect of the Cu Source on Optical Properties of CuZnO Films Deposited by Ultrasonic Spraying

**DOI:** 10.3390/ma7021261

**Published:** 2014-02-18

**Authors:** Chih-Hung Hsu, Lung-Chien Chen, Xiuyu Zhang

**Affiliations:** Department of Electro-Optical Engineering, National Taipei University of Technology, No.1, Section 3, Chung-Hsiao E. Rd., Taipei 10608, Taiwan; E-Mails: bluerex6@ms35.hinet.net (C.-H.H.); zxy.1013@live.com (X.Z.)

**Keywords:** CuZnO film, copper source, optical properties

## Abstract

CuZnO (CZO) films have received considerable attention, owing to their potential applications in semiconductor devices, including gas sensors or solar cells. However, exactly how these films affect the properties of CZO films by using different Cu sources has seldom been investigated. This study demonstrates the feasibility of preparing CZO films by using different Cu sources via a simple ultrasonic spray method, in which copper nitrate and copper acetate were used as copper sources. Optical properties of CZO films prepared by copper nitrate and copper acetate were also investigated, based on transmittance and photoluminescence measurements. Additionally, the composition and the morphology of the films were investigated using the X-ray diffraction analysis and field emission scanning electron microscopy. The results of this study demonstrate that the CZO films prepared by using copper acetate exhibit better optical properties.

## 1. Introduction

As a wide direct bandgap (3.36 eV) II-VI compound semiconductor, zinc oxide (ZnO) has a large exciton binding energy of 60mV with a hexagonal wurtzite structure [1]. However, copper oxide is a semiconductor with a band gap of 1.4 eV, which absorbs strongly in a visible spectrum [2]. CuZnO (CZO) with different forms (e.g., thin films, nanorods and powder which is combined by ZnO and CuO) has been extensively studied, owing to its potential applications in many fields, including gas sensors and solar cells [3–9]. Many studies have focused on the change of physical properties when Cu was induced into ZnO. Caglar *et al*. [10] examined the tunable optical and electrical properties of CZO films which were prepared by the sol-gel spin coating method. Shaema *et al*. [11] investigated the room-temperature ferromagnetic behavior of CZO nanorods. Several copper sources, including copper nitrate, copper acetate and copper chloride, have been used to prepare CZO films via the chemical pyrolysis method [12–14]. However, exactly how copper sources affect the physical properties of CZO films has seldom been addressed.

In this study, owing to its high growth rate, large area uniformity, and inexpensive preparation, the ultrasonic spray method is adopted to prepare CZO films by using different Cu sources [14,15]. The structures, morphologies, and properties are measured using various approaches.

## 2. Results and Discussion

### 2.1. Crystalline Structure

**Figure 1.**
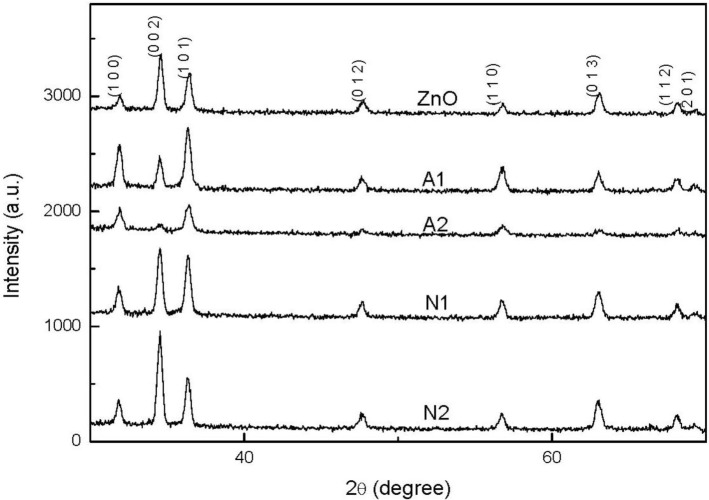
XRD patterns of ZnO and CuZnO (CZO) thin films using different copper sources (Reference code: JCPDF # 75-0576 for ZnO).

Figure 1 shows the XRD patterns of ZnO and CZO films deposited on glass substrates. The spectra in this study reveal broad peaks at the position of 31.86°, 34.57°, 36.39°, 47.66°and 56.71°, which correlate well with (100), (002), (101), (012), and (110) planes of the ZnO phase. This finding suggests that the thin film is polycrystalline and has a hexagonal wurtzite structure of ZnO phase. The peaks observed above closely correspond to the hexagonal wurtzite structure ZnO (JCPDF # 75-0576). This figure also reveals that the peak intensity decreases with an increasing Cu concentration increased and the use of copper acetate. However, when copper nitrate is used, the intensities of (002) and (013) planes increase with an increasing Cu concentration. The difference in (100) and (002) planes between samples A1 and N1 implies the formation of different crystalline orientations by using different copper sources, which is also confirmed by the following SEM analysis. The strongest intensity for the samples A1 and N1 are (101) and (002) planes, respectively. In order to attain the detailed structure information, the grain size *G* along the c-axis was calculated according to the Scherrer’s equation [16]:

$$G=\frac{0.9\lambda }{\beta \text{cos}\theta }$$ (1)

where *G*, ë, â, and è denote the grain size, the X-ray wavelength, the full width at half maximum (FWHM) in radians, and the Bragg angle of (002) or (101) peak, respectively. The grain size for the samples A1 and N1 are 44.1 and 43.9 nm, respectively. Therefore, the crystallinity of the sample A1 using copper acetate is better slightly than that of the sample N1. In contrast to the peaks of ZnO film, the (002) peak of CZO films shifts slightly from 34.62 to 34.5°, indicating that the Cu atoms have replaced the Zn atoms in the films.

**Figure 2.**
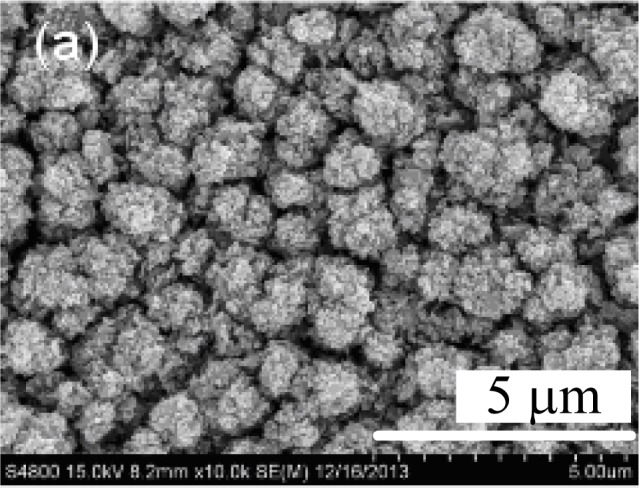

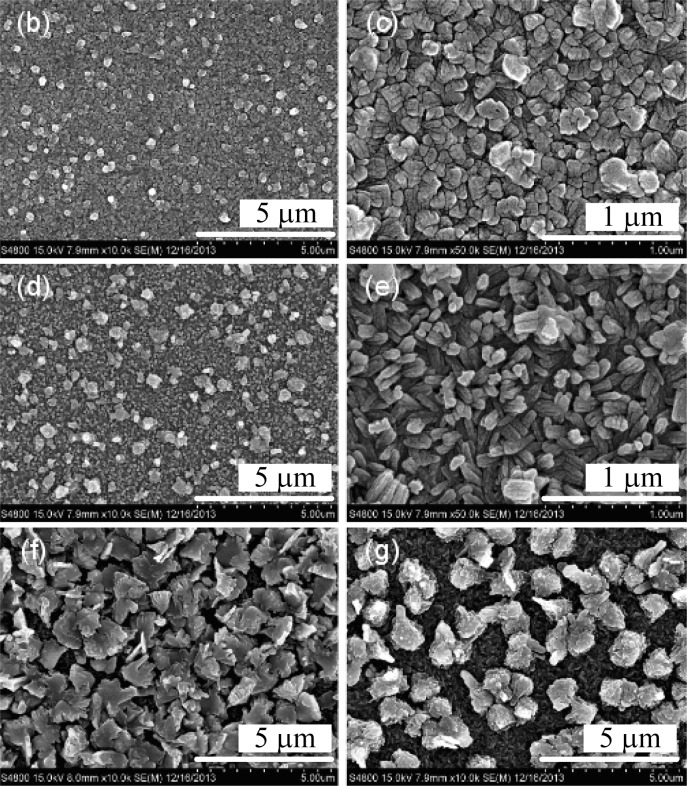
Field emission scanning electronic microscopy (FESEM) images of ZnO and CZO films with different magnification: (**a**) ZnO with 10 k magnification, A1 with (**b**) 10 k magnification and (**c**) 50 k magnification, A2 with (**d**) 10 k magnification and (**e**) 50 k magnification, (**f**) N1 and (**g**) N2 with 10 k magnification.

Figure 2 depicts the field emission scanning electronic microscopy (FESEM) images of the surface morphologies of ZnO and CZO films observed. According to this figure, the films using different copper sources significantly differ from each other in morphology. Large rods with a diameter of 2 μm are observed on the surface of the ZnO film. According to Figure 2b,d, the diameter of small rods on the surface of CZO films prepared by using copper acetate ranges from around 0.5–1 μm. Additionally, the diameter and density of rods increases with an increasing Cu concentration. However, when copper nitrate is used, the morphology transforms from a sheet structure to aggregative rods. The size of sheet structures shown in Figure 2f is approximately 2.5 μm, while the diameter of a single rod shown in Figure 2g is about 1.5 μm. The morphology structure of the films changes in a regular pattern as the copper acetate used while it changes significantly when copper nitrate is used. The different morphology structures between samples prepared by different copper sources (correlate well with the differences in their XRD patterns. The SEM images indicate that the films are both polycrystalline, which corresponds to the XRD analysis results and also implies the difficulty in controlling the morphology of the film when copper nitrate is used.

### 2.2. Optical Characteristics

**Figure 3.**
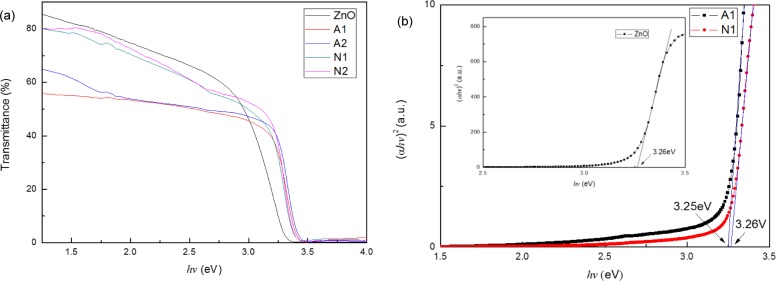
Transmittance and optical band-gap properties of the ZnO and CZO films: (**a**) optical transmission spectra of the ZnO and CZO films; (**b**) the graph of (α*hv*)^2^ *vs. hv* plots for the CZO films prepared by using copper acetate and copper nitrate and the ZnO film (inset).

Figure 3a shows the optical transmittance spectra of ZnO and CZO films ranging from 1.5 eV (827 nm) to 4 eV (310 nm). The films prepared by using copper nitrate have a higher transmittance ranging from 1.5 eV to 3.25 eV, indicating that the films have a better transparency in the visible region than the ones prepared by using copper acetate. However, when the Cu concentration increases and the copper source is fixed, the transmittance of the films remains nearly constant. This observation differs from that reported in [10] and may be attributed to the preparation method. Although the ratio of Cu to Zn ions in the prepared films is determined by the precursor solution, controlling the exact reacting ratio is rather difficult. When the Cu concentration increases in the precursor solution, the amount of Cu ions incorporated into the films may not increase high enough to influence the transmittance.

The direct optical band gap E_g_ of the films (Figure 3b) is obtained from the transmission spectra by using the following relationships:

αd=−lnT (2)

$${\left(\alpha hv\right)}^{2}=A(hv-E_{g})$$ (3)

where α is the absorption coefficient values; *d* is the film thickness; *T* refers to the transmittance spectra of thin films; *A* denotes a constant; *hv* represents the photon energy and *E_g_* is the optical band gap of the semiconductor.

Figure 3b shows the graph of α^2^ versus *hv* plots for the film using copper acetate and copper nitrate. This figure reveals that the extrapolation of the linear portion of the graph to energy axis at (α*hv*)^2^ = 0 produces the *E*_g_ value. The optical band gap of the films is quite close to each other. The optical band gap of A1, which is prepared by using copper acetate, is 3.25 eV; meanwhile, N1, which is prepared by using copper nitrate, is 3.26 eV. The minute difference of optical band gap may be owing to the difference of the morphology structure [15], because ZnO particles with different shapes have different crystalline defect contents, subsequently impacting the *E_g_* values [17]. We can infer that different Cu sources negligibly affect the optical band gap of prepared CZO films.

**Figure 4.**
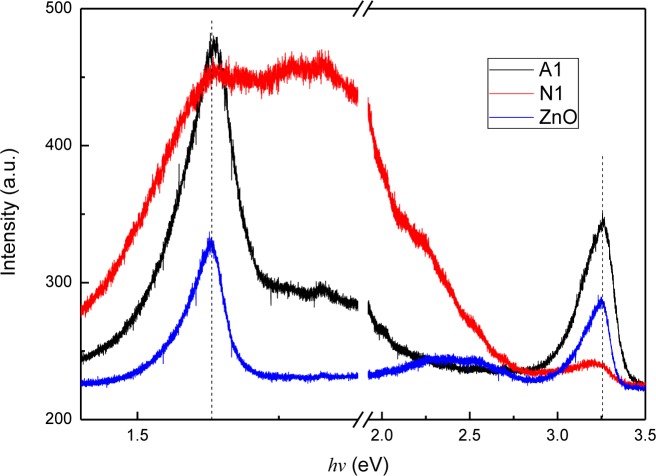
Room temperature photoluminescence (PL) spectra of ZnO and CZO films. The vertical dashed lines denote the peaks position of near-band-edge (NBE) and the emission caused by oxygen vacancy.

Photoluminescence (PL) measurement of the ZnO and CZO films was conducted to accumulate further information of optical properties. Figure 4 shows the PL spectra of the films. Peaks of the films are both appear at 3.26 eV (380 nm) and 1.63 eV (760 nm). The peak at 3.26 eV corresponds to the optical band gap of the wide band gap CZO films and can be attributed to the recombination of free excitons through an exciton-exciton collision process [18,19]. Many investigations have posited that the near-IR emission at about 760 nm originates from defects similar to those responsible for the red emission in ZnO, *i.e.*, oxygen vacancy [20,21]. However, some studies have ascribed the near-IR peak to the second order diffraction of the NBE emission [22–25]. While considering the high intensity of the near-IR peak and the copper doping, we believed that the peak at 1.63 eV is attributed to the oxygen vacancy. Additionally, both broad emission peaks ranging from 1.75 eV (709 nm) to 2.25 eV (551 nm) are observed, and the intensity of the film prepared by nitrate is stronger than that of the film prepared by acetate. Moreover, the broad emission is related to the deep-level emission, which is caused by the electron transformed from zinc interstitial (*Zn_i_*) to oxygen vacancy (*V*_o_) defect levels [26]. Moreover, the PL spectrum of A1, which is prepared by using copper acetate, shows higher intensity of NBE emission and lower intensity of deep-level emission than those of N1, indicating that the film using copper acetate as copper source has better crystallinity.

**Figure 5.**
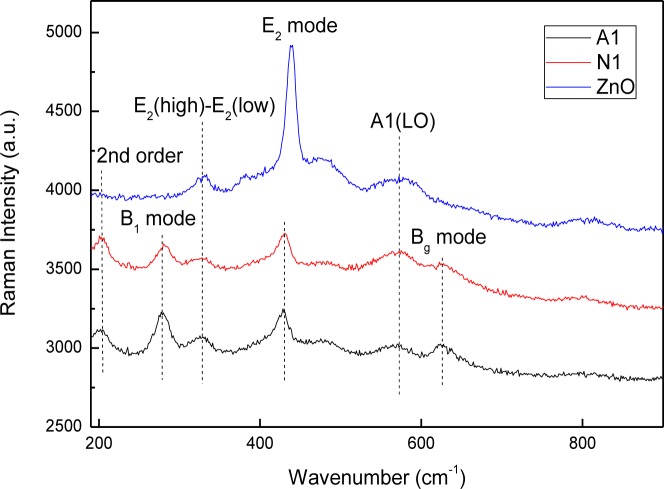
Room-temperature Raman spectra of ZnO and CZO films. The spectra are vertically offset for clarity. The E_2_ mode shifts when Cu is incorporated into the films.

This study also investigated how copper doping affects the microscopic structure and vibration properties of prepared CZO films, based on Raman spectroscopic studies and a comparison with the ZnO film. Figure 5 shows the room-temperature Raman spectra of ZnO and CZO films. The Raman modes at 203 cm^−1^, 428 cm^−1^ and 570 cm^−1^ can be attributed to the second order vibrations, E_2_ mode and A_1_(LO) mode, owing to the published data for c-ZnO respectively [27–29]. Additionally, the A_1_(LO) mode is attributed to an electrical field induced (EFI) scattering, which also induces the inactive B_1_ mode at 277 cm^−1^ [30,31]. The modes at 624 cm^−1^ can be ascribed to the B_g_ mode of CuO phase [32,33]. The mode at 328 cm^−1^ should be related to the E_2_(high)-E_2_(low) mode of ZnO phase [30,34]. Comparing the spectra of CZO films with those of ZnO films reveals that the peak intensity of E_2_ mode decreases and the position shifts when Cu is incorporated into the films, and B_1_ and B_g_ modes generate. The two similar spectra reveal that the microscopic structure and vibration properties of the films prepared by using different Cu sources resemble each other as well.

## 3. Experimental Details

CZO films were deposited by ultrasonic spraying method at atmospheric pressure on a glass substrate. Prior to deposition, the glass substrates were first cleaned in acetone and methanol. The substrates were then rinsed in de-ionized water and dried in the flowing nitrogen. Notably, the precursor solution was obtaining by selecting zinc acetate as the source of zinc, copper acetate or copper nitrate as the source of copper, respectively. Ammonium acetate was used as the *p*-type dopant nitrogen source, and the carrier concentration in the CZO films was around (3–15) × 10^19^ cm^−3^, as estimated by Hall measurements. Based on our experiences, ammonium acetate greatly facilitates the deposition of the film and improves the crystalline quality of the film. Without using ammonium acetate, depositing the film would be extremely difficult and the crystalline quality would be inferior. The molar concentration of ammonium acetate solution was 0.45 M, and the reagents of copper source (copper acetate or copper nitrate) and the zinc acetate was 0.15 M altogether. The samples marked as A1 and A2 were prepared by using copper acetate as the copper source; the relative Cu and Zn concentrations of the precursor solutions are 1:9 and 2:8, respectively. Although the samples marked as N1 and N2 have the same relative Cu and Zn concentration as above, in this study, copper nitrate was used as the copper source. The solutions were then stirred at room temperature for 1 h and then moved into a commercial ultrasonic nebulizer where makes the solutions be aerosol. The aerosol was transported to the substrate by high-purity nitrogen gas, and the substrate was kept at 500 °C. The deposition time lasted for 15 min. ZnO film was also prepared for reference by undergoing the same process using a precursor solution without the copper source.

The crystalline microstructure of the films was determined by a PANalytical X’Pert Pro DY2840 X-ray diffraction with Cu-Kα radiation (λ = 0.1541 nm) in the scanning range between 2θ = 30° and 70°. Surface morphology was studied by a LEO 1530 field emission scanning electron microscope (Zeiss, Jena, Germany). Optical transmission properties were measured in the range of 350–900 nm by using a Hitachi U-2001 Ultraviolet-Visible spectrophotometer (Hitachi, Tokyo, Japan). Photoluminescence (PL) spectra were excited by a He-Cd laser (325 nm), and Raman spectra were excited by a second-harmonic line of Nd^3+^ laser (532 nm), which was both collected by a Dongwoo spectrophotometer (Dongwoo, Soule, Korea) at room temperature.

## 4. Conclusions

This study elucidated the structural, morphological, and optical properties of CZO films by using different copper sources prepared via the ultrasonic spray method. The XRD patterns indicated that the films are both polycrystalline. The transmission measurement and the Raman spectra of the films revealed that the cooper sources only slightly affect the optical band gap, microscopic structure and vibration properties. The films both showed strong Near-IR emissions of photoluminescence features. Meanwhile the intensity difference of broad deep-level emission indicates that the CZO films prepared by using copper acetate have a better crystallinity than that of the film prepared by copper nitrate. Results of this study demonstrate that the prepared CZO films using copper acetate have better crystalline and optical properties than those using copper nitrate.
